# Implementation of a Newborn Clinical Decision Support Software (NoviGuide) in a Rural District Hospital in Eastern Uganda: Feasibility and Acceptability Study

**DOI:** 10.2196/23737

**Published:** 2021-02-19

**Authors:** Mary Muhindo, Joshua Bress, Rogers Kalanda, Jean Armas, Elon Danziger, Moses R Kamya, Lisa M Butler, Theodore Ruel

**Affiliations:** 1 UCSF Preterm Birth Initiative University of California San Francisco School of Medicine San Francisco, CA United States; 2 Infectious Diseases Research Collaboration Kampala Uganda; 3 Global Strategies Albany, CA United States; 4 School of Medicine Makerere University College of Health Sciences Kampala Uganda; 5 Institute for Collaboration on Health, Intervention and Policy University of Connecticut Storrs, CT United States; 6 Department of Pediatrics University of California, San Francisco San Francisco, CA United States

**Keywords:** clinical decision support, neonatology, neonatal mortality, mHealth, mobile phone

## Abstract

**Background:**

Lack of trained health care workers and nonadherence to national guidelines are key barriers to achieving high-quality newborn care in health care facilities in low- and middle-income countries. Traditional didactic approaches addressing these barriers fail to account for high staff turnover rates and result in temporary behavior change. NoviGuide, a clinical decision support software designed to standardize neonatal care through point-of-care assessments, has the potential to align bedside practice to national guidelines in settings lacking subspecialty neonatal providers.

**Objective:**

This study aims to determine the adaptation, adoption, feasibility, acceptability, and sustainability of NoviGuide and its impact on nurse-midwives’ knowledge in a rural hospital in eastern Uganda.

**Methods:**

This mixed methods observational study was guided by the Proctor framework. Experts reviewed the clinical content of NoviGuide to ensure fidelity to Uganda guidelines. We enrolled nurses and midwives providing newborn care at Tororo District Hospital, trained them on NoviGuide use, and followed them for 12 months. We assessed adoption, feasibility, acceptability, and sustainability by analyzing NoviGuide use data, comparing it with maternity registry data and administering the System Usability Scale (SUS) and the Center for Health Care Evaluation Provider Satisfaction Questionnaire. We compared the mean knowledge assessment score at baseline, 6 months, and 12 months using a two-tailed *t* test.

**Results:**

Five Ugandan experts suggested two minor changes to NoviGuide: the inclusion of an unsterile birth environment as an indication for empiric antibiotics and the addition of a reminder to follow-up with newborns with temperatures between 37.7°C and 37.9°C. Of the 19 nurse-midwives enrolled in February 2017, 74% (n=14) completed the follow-up in March 2018. The participants entered a total of 1705 assessments of varying newborn characteristics into NoviGuide throughout the day, evening, and night nursing shifts. The SUS score at the end of the study was very high (93.5, above the average of 68). Participants had a positive perception about NoviGuide, reporting that NoviGuide saved time (mean 5, SD 0) and prevented mistakes (mean 5, SD 0), and that they felt more confident in taking care of newborns when they used NoviGuide (mean 5, SD 0). Participants were highly satisfied with NoviGuide (mean 4.86, SD 0.36), although they lacked medical supplies and materials needed to follow NoviGuide recommendations (mean 3.3, SD 1.22). The participants’ knowledge scores improved by a mean change of 3.7 (95% CI 2.6-4.8) at 6 months and 6.7 (95% CI 4.6-8.2) at 12 months (*P*<.001).

**Conclusions:**

NoviGuide was easily adapted to the Uganda guidelines. Nurse-midwives used NoviGuide frequently and reported high levels of satisfaction despite challenges with medical supplies and high staff turnover. NoviGuide improved knowledge and confidence in newborn care without in-person didactic training. NoviGuide use has the potential to scale up quality newborn care by facilitating adherence to national guidelines.

## Introduction

### Background

In 2018, 2.5 million children died in their first 28 days of life worldwide, with the highest neonatal mortality rate observed in sub-Saharan Africa (28 per 1000 live births) [1]. The causes of death in the neonatal period are well known, including complications of prematurity; intrapartum complications; and infections, such as sepsis, pneumonia, and meningitis [2]. Neonatal care protocols, such as those described in the World Health Organization (WHO) Essential Newborn Care guidelines [3], can lead to significant improvements and comprise the core of current global efforts to reduce neonatal mortality [4-7]. However, lack of trained health care workers and nonadherence to national neonatal care clinical guidelines are key barriers to achieving high-quality newborn care in health care facilities in low- and middle-income countries (LMICs) [8]. Implementation of evidence-based protocols in LMICs has been hampered by significant challenges: in-person training does not reliably lead to changes in workplace behavior, medical errors are common, changes in practice are lost quickly without reinforcement, high staff turnover rate, and performance of health care providers is difficult to monitor [9-12].

Traditional approaches to implementing neonatal care clinical guidelines in LMICs are based on lectures, the distribution of educational material, and hands-on training; although these approaches can be effective for focused topics or procedures such as neonatal resuscitation [13,14], they are not well suited to multistep neonatal protocols. A key component of behavior change is immediate and consistent feedback. Health care providers learning neonatal resuscitation receive immediate feedback on their performance through a newborn’s second-to-second response to care, whereas the degree to which a provider’s care aligns to a complex neonatal protocol, such as an antibiotic prescription guideline, cannot anticipate a similar deliberate practice benefit [15]. In addition, although the performance of a hands-on procedure is relatively constant across diverse patients, the application of complex neonatal protocols requires that health care providers must often account for patient-specific factors and adjust for site-specific constraints [16].

Clinical decision support (CDS) software has the potential to enable health care providers to deliver complex medical protocols as responsive point-of-care assessments [17-21]. CDS aims to achieve desired quality aims through care standardization rather than relying on individual performance, while improving health care provider satisfaction by deploying medical protocols through a validated user interface. A key feature that distinguishes CDS from traditional didactic training is that it does not rely on the health care provider to summon previously learned content at the point-of-care, either from memory or by seeking a resource. Instead, CDS uses patient-specific factors, such as a patient’s age, vital signs, and symptoms, and directs health care providers to potentially relevant medical protocols.

### NoviGuide

Our team developed NoviGuide (Global Strategies) [22], a tablet-based CDS software, to optimize the facility-based care of newborns in LMICs by transforming complex neonatal disease-specific protocols into comprehensive patient assessments.

**Figure 1:**
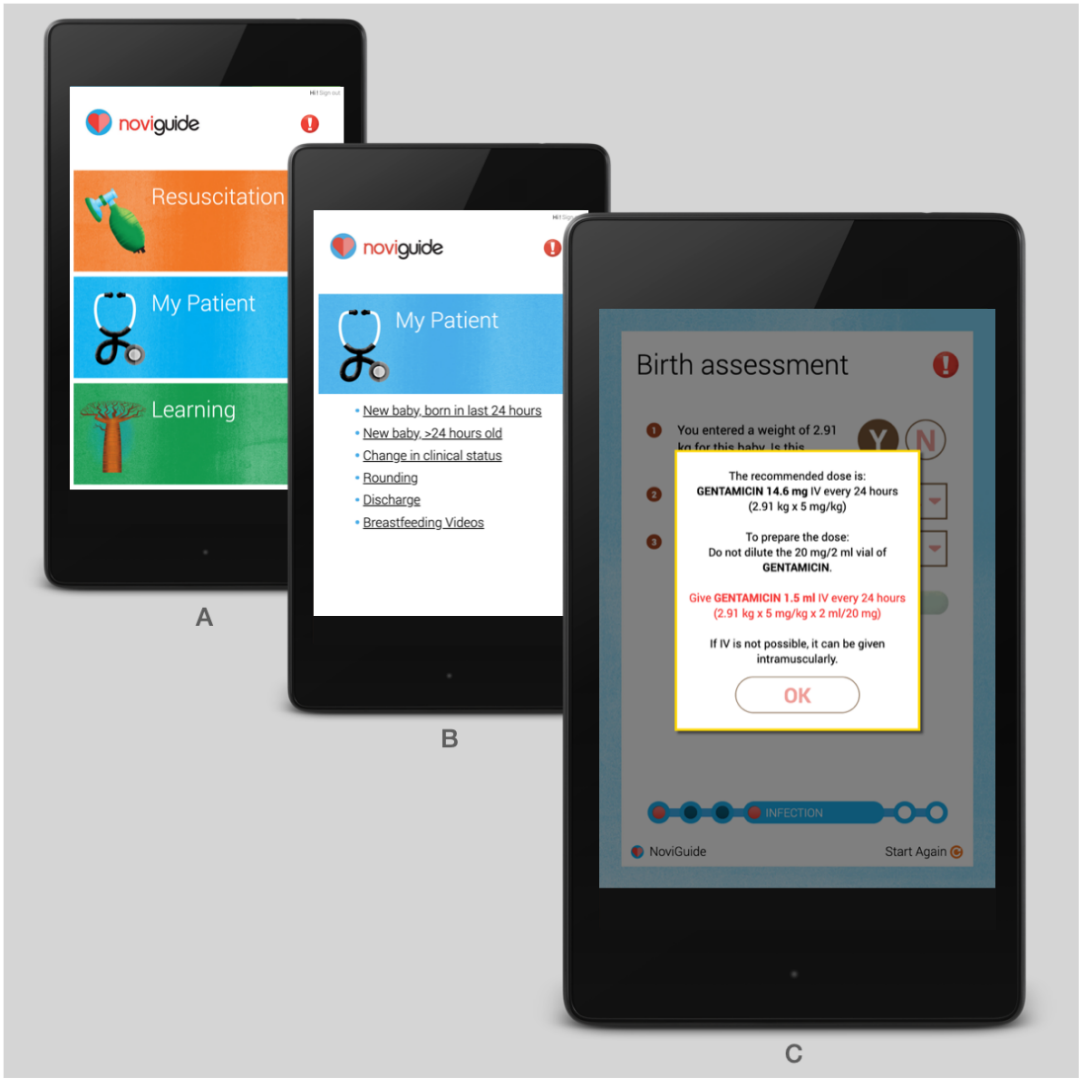
NoviGuide screenshots: Home page, NoviGuide clinical assessments, and Medication dosing instructions pages.

NoviGuide has 3 main sections: *Resuscitation*, *My Patient*, and *Learning* (Figure 1). The *Resuscitation* section contains an instructional three-dimensional animation depicting the steps of neonatal resuscitation (Multimedia Appendix 1). The *My Patient* section houses point-of-care CDS algorithms that guide health care providers through the initial assessment and daily care of newborns. The *Learning* section is a standard e-book with additional information on neonatal topics and videos from the Global Health Media Project [23] depicting physical signs, common newborn procedures, and breastfeeding positions. A menu bar includes an *emergency* button to directly access clinical guidance for the treatment of neonatal seizures and abdominal emergencies (Figure 1). We selected algorithms for the emergency section based on the need for rapid guidance and the ability to generate that guidance based solely on the current dosing weight. NoviGuide was created by the nonprofit organization Global Strategies [24] for widespread use in low-resource settings. Global Strategies built the core software and content of NoviGuide and partnered with Plexus Medical Arts [25] to design the resuscitation video. The Global Health Media Project allowed the inclusion of its neonatal and breastfeeding films in the software.

The *My Patient* CDS content includes 4 comprehensive clinical assessments to assist health care providers in the initial assessment; subsequent care; and discharge of well, sick, and preterm newborns. These assessments are named *new baby more than 24 hours old or change in clinical status, rounding*, and *discharge* (Figure 1)*.* The assessments provide step-by-step prompts to guide the health care provider to enter data from the physical examination findings and key pieces of medical history (Multimedia Appendix 2). The assessments are dynamic, adding more questions in response to danger signs and alerting users to potential inconsistencies in their responses. NoviGuide then makes case-specific management recommendations derived from national clinical guidelines that are tailored to the newborn’s weight, gestational age, day of life, clinical features, and available equipment. The *My Patient* assessments include numerous job facilitators to incentivize use, save time, and decrease medical errors (Multimedia Appendix 2). These job facilitators include preterm feeding calculators, medication and fluid calculators, guidance interpreting vital signs, and the generation of a printable summary. Responses to abnormal vital signs, infection risk stratification for antibiotic use, medication doses, and fluid calculations are provided automatically alongside any equations used to derive the recommendations (Figure 1).

When initiating an assessment, health providers are asked to indicate if the assessment is being completed on a *real baby* or *just practicing* (Multimedia Appendix 2). Users are often prompted to consider actions in NoviGuide’s pathways that require resources. In numerous instances, users can indicate if they encounter a resource constraint related to the guidance, and these data are captured. NoviGuide works either offline or when connected to WiFi. All the information contained in the NoviGuide is present in the initial download. When NoviGuide is connected to the internet by Wi-Fi, data are automatically synchronized to a cloud-based database. NoviGuide is designed to work on Google Android, iOS, and FireOS platforms.

With this study, our aim is to describe the adaptation, adoption, feasibility, acceptability, and sustainability of NoviGuide use in a rural district hospital in eastern Uganda. We used a mixed methods observational study design among nurses and midwives in the context of newborn care. In addition, we analyzed the impact of NoviGuide use on the knowledge of nurse-midwives. The Proctor framework [26] guided the definition of implementation constructs with respect to the users and the local context.

## Methods

### Adaptation of NoviGuide to Uganda Clinical Guidelines

With the assistance of the Uganda Pediatrics Association, we recruited a team of 5 Ugandan experts to review the content of NoviGuide over a series of meetings between August and November 2016. The aim was to ensure the fidelity of NoviGuide to the Uganda neonatal care clinical guidelines and to refine the NoviGuide design to suit the Uganda local context [27]. The study team presented an overview and instructions on how to visualize and verify the content, order, and branch-point logic of NoviGuide’s decision trees (Table 1). The study team also showed the experts how to evaluate NoviGuide’s decision trees using test cases comprising sick and well neonatal scenarios. The study team highlighted specific content in which there are frequent variations in recommendations among national guidelines, including the preparation of medications and various pharmacologic diluents, the management of well-appearing newborns born to mothers with fevers and/or other sepsis risk factors, and the threshold at which a glucose level is considered low. For each clinical area, the experts viewed a series of videos that explained the clinical topic and how it manifests in NoviGuide’s *My Patient* assessments. The study team gave each expert a tablet (Amazon Fire HD 8) loaded with NoviGuide version 1.6 to take home and instructed them to make notes of any recommendations. The tablets were returned following this activity. The Ugandan experts met for a second meeting for free discussion of their findings and to achieve consensus on a set of recommendations for Global Strategies.

**Table 1:** Description of content areas for Ugandan expert panel review.

Area	Description
Content	Verifying that the clinical information is consistent with national protocols. For example, the study team shows the expert panel the temperature threshold where a newborn is considered febrile. The expert panel then votes to confirm or modify.
Order	Verifying that the order of questions in each assessment is consistent with national protocols. For example, confirming that lines of questioning concerning hypoglycemia should precede lines of questioning concerning the initiation of antibiotics.
Branch-point logic	Reviewing how the software responds to user input and verifying that the response is consistent with national protocols. For example, the study team shows the expert panel the alert message a user sees after entering a risk factor for infection. The expert panel then votes to confirm or modify.

### Study Setting

We conducted an implementation study from February 2017 to March 2018 at Tororo District Hospital (TDH), a rural government-owned district hospital in eastern Uganda. TDH, a 200-bed facility, serves approximately 517,000 people, the majority of whom live in rural areas [28]. The hospital conducts about 360 births per month and receives sick newborns from the community, surrounding health centers, and private facilities including referrals from across the Kenya-Uganda border. One medical officer is assigned to work in the maternity ward. Nurses and midwives provide the majority of newborn care working in shifts of 2 to 3 providers. In addition, these same nurses and midwives provide care for laboring women and postpartum mothers and conduct all vaginal deliveries in a 6-bed labor suite. TDH has basic newborn care supplies, including bag-valve-masks, warming tables for resuscitation, intravenous (IV) supplies, and a standard country formulary for medications. At the time of the study, TDH did not have a dedicated neonatal care unit. Newborns requiring close nursing attention were cared for on 2 warming tables within the labor suite. Newborns requiring only intermittent IV antibiotic therapy were admitted by the medical team in the general postpartum ward with women requiring postnatal care.

### Enrollment and Training of Study Participants and Launch of Study

We screened all the nurses and midwives working in the maternity ward at the TDH and enrolled them into the study in February 2017. Inclusion criteria for nurse-midwives included providing newborn care at TDH, having current licenses to practice, and willingness to participate in the study. In addition, nurse-midwives had to have completed the WHO Integrated Management of Childhood Illnesses modules [29]. Participants exited the study if they were transferred by the hospital administration to another ward within the hospital, ceased employment at the hospital, or withdrew consent.

To recruit participants, we invited all nurse-midwives working at the TDH and their supervisors to attend an organized meeting in the hospital boardroom. The study team provided a brief introduction about the study, including NoviGuide and evaluation methods, and obtained written informed consent from the nurse-midwives who met the eligibility criteria. We asked the nurses and midwives who declined participation for their reasons. The medical superintendent, matron, and wards-in-charge were recruited as key informants in the study development; hospital leadership encouraged but did not mandate or require the use of NoviGuide.

Following enrollment, the study participants attended a 3-hour training conducted by representatives of Global Strategies on how to use NoviGuide. Following the training, the participants created individual unique usernames and passwords to log in to the tablet and the NoviGuide software. The study team provided 7 tablets (Amazon Fire HD 8 tablet) loaded with NoviGuide in February 2017. The tablets were stored in a lockable wooden cabinet in the nurses’ office in the labor suite. During the first week, the study team provided on-site technical support to troubleshoot technical issues. We followed the study participants through March 2018.

### Data Collection

At baseline, participants completed a survey that included demographic data (age, sex, and level of education), years of clinical experience, experience using technology, and perceived challenges in caring for newborns at TDH.

The study participants also completed a questionnaire assessing basic knowledge in newborn care, including questions about the management of hypoglycemia, indications for antibiotics, management of the HIV-exposed infant, and the specific order of tasks in neonatal resuscitation. This questionnaire was then repeated at 6 and 12 months with modifications of the question order and variables, such as newborn weights, in the clinical scenarios (Multimedia Appendix 3).

Throughout the study period, the study team connected the tablets to a Wi-Fi network once per day to upload NoviGuide use data, stored in the tablet, onto a secure cloud-based database. NoviGuide use data were linked to the participants’ unique study identification number. We compared the total number of assessments entered into the NoviGuide with the total number of births and admissions of newborns at the hospital during the study period. The study team instructed the participants to keep notes on any technical problems encountered during NoviGuide use in a study logbook or contact the study team by SMS, phone, or email for urgent concerns.

At 12 months, the participants completed 2 validated measures of software usability. We used the System Usability Scale (SUS) [30], consisting of 10 standard questions, where a statement is made and the respondent then indicates the degree of agreement or disagreement with the statement in a Likert scale format with responses 1 to 5, where 1 represents strongly disagree and 5 represents strongly agree. We also used a provider satisfaction questionnaire adapted from the Center for Health Care Evaluation Provider Satisfaction Questionnaire (CHCE-PSQ) [31], which has 8 questions and the respondent then indicates the degree of agreement or disagreement with the statement in a Likert scale format with responses 1 to 5. For questions 1 to 4, response 1 represents poor and 5 represents excellent. Whereas for questions 5 to 8, response 1 represents strongly disagree and 5 represents strongly agree. In addition, participants completed an end-of-study questionnaire containing 15 questions assessing perceived acceptability and feasibility of NoviGuide using a Likert scale of 1 to 5, where 1 represents strongly disagree and 5 represents strongly agree (Multimedia Appendix 3).

### Analysis

We defined adoption as the measure of the initial uptake or intention to use the NoviGuide and measured it by reviewing the NoviGuide use data for (1) the different assessments made into the NoviGuide and how many of these were completed through to the summary page, (2) the time participants spent during the NoviGuide assessments, (3) NoviGuide use during the different nursing shifts (day, evening, and night), (4) whether participants accessed the NoviGuide’s educational videos or reading materials and whether the participants used the NoviGuide for practice or with a real newborn, and (5) total NoviGuide assessments in relation to the total births and admissions at the hospital during the study period.

We defined acceptability as the measure of the participants’ satisfaction with the various components of NoviGuide, including content, complexity, navigation, ease of use, and general experience using NoviGuide for newborn care, and measured it by (1) comparing the overall SUS score with an average score of 68, as described by John Brooke [30], and (2) determining the mean scores and SD of the questions in the CHCE-PSQ and end-of-study questionnaire. We calculated the overall SUS score by summing up the score contributions of each question and multiplying it by 2.5.

We defined feasibility as the actual fit and the use of NoviGuide within the rural hospital context and measured it by reviewing the NoviGuide use data for (1) the characteristics of newborns cared for using NoviGuide and (2) whether the study participants indicated resource or health system constraints that could prevent the use of NoviGuide. We also measured feasibility by determining the mean scores and SD of questions 11 to 15 of the end-of-study questionnaire assessing the availability of medical supplies and materials needed to follow NoviGuide recommendations; time to use the NoviGuide; and support from colleagues, supervisors, and hospital administrators.

We defined sustainability as the extent to which NoviGuide use was maintained throughout the study period and the frequency and degree of technical problems preventing NoviGuide use. We measured use over the study period by individual users and collectively, across 100-day interval study periods (day 0-99, 100-199, 200-299, and 300-397).

We measured the impact of NoviGuide use on participant knowledge by comparing the mean knowledge assessment score at baseline with scores at 6 and 12 months using a paired *t* test. We used Stata (version 16, StataCorp) for all statistical analyses. A *P* value of <.05 was considered significant.

### Ethical Review

The University of California San Francisco Committee on Human Research (16-19241), the Makerere University School of Biomedical Sciences (SB-352), and Uganda National Council for Science and Technology (IS 125) approved the study. All study participants provided written informed consent before participation in the study-related activities.

## Results

### Adaptation of NoviGuide to Uganda Clinical Guidelines

The study team selected 4 Ugandan neonatologists and 1 Ugandan neonatal nurse as expert reviewers. The experts suggested 2 modifications to the decision trees. First, they recommended that birth in an unsterile environment should be added as a sepsis risk factor and that its presence should prompt a recommendation for empiric antibiotics. Second, they recommended that a specific pop-up message be generated for temperatures between 37.7°C and 37.9°C to alert users that the newborn was *warm* and they suggested that a follow-up temperature measurement be taken. Global Strategies incorporated these modifications into the NoviGuide decision trees.

### Participant Characteristics and Follow-Up

**Figure 2:**
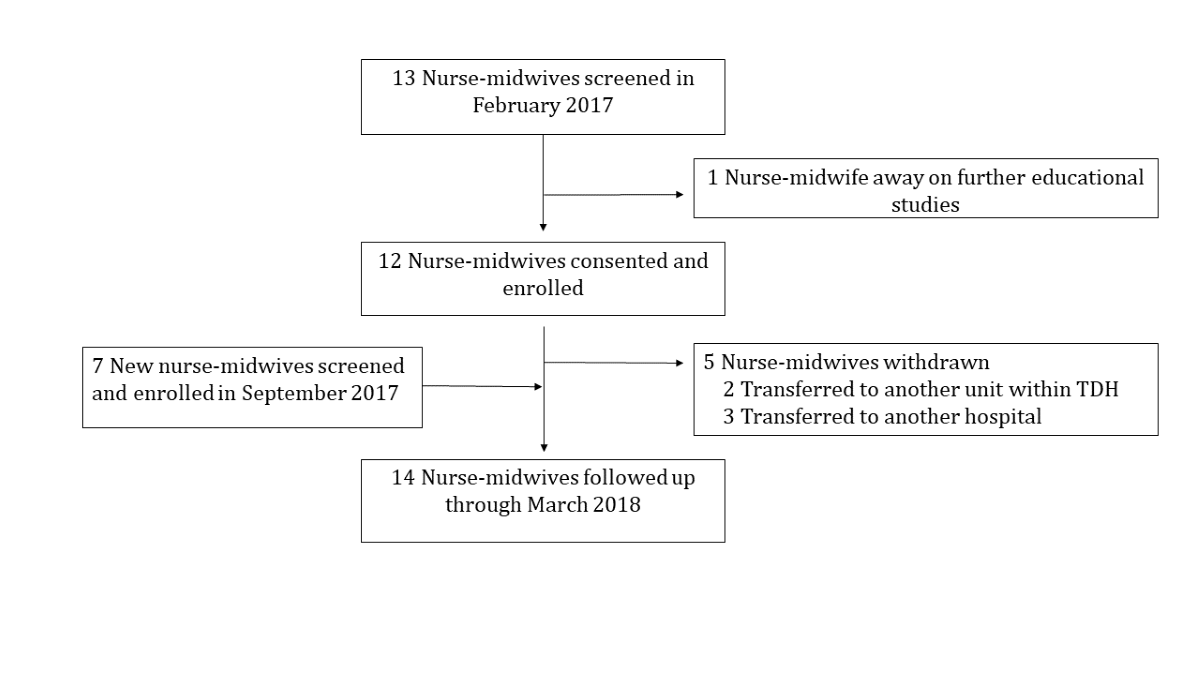
Study flow diagram. TDH: Tororo District Hospital.

The study team screened 13 nurse-midwives and enrolled 12 nurse-midwives in February 2017 (Figure 2). One nurse declined to participate, citing that she was going to be away for further educational studies. Of the 12 participants in the initial enrollment group, 1 had a late start date of May 2017 because of maternity leave, and 5 participants were transferred to either other units within TDH or to other hospitals during the study period. In September 2017, following new hires at the maternity ward, the study team screened and enrolled an additional 7 nurse-midwives as replacements for those who had been transferred from the maternity ward. All 19 (100%) study participants were female, with a mean age of 39 (SD 14) years (Table 2). Of the 19 participants, 11 (58%) reported using a calculator on their phones to calculate medication dosages and fluid rates, 3 (16%) used a handheld calculator, and 5 (26%) performed the calculations mentally. All 19 participants reported that they believed that technology could help them in the care of newborns. Of the 19 enrolled participants, 14 (74%) were followed up with until the end of the study period.

**Table 2:** Baseline demographics and participant characteristics (n=19).

Characteristics			Values
Female, n (%)			19 (100)
Age (years), mean (SD)			39 (14)
**Highest educational level, n (%)**			
	Bachelor’s	1 (5)	
	Registered nurse	12 (63)	
	Certified midwife	6 (32)	
**Work experience (years), n (%)**			
	0-2	4 (21)	
	3-10	5 (26)	
	11-20	4 (21)	
	>21	6 (32)	
**Devices owned personally, n (%)**			
	None	2 (11)	
	Home computer or laptop	1 (5)	
	Tablet	0 (0)	
	Smartphone	9 (47)	
	Ordinary phone	9 (47)	
**How frequently do you access the internet, n (%)**			
	Never	5 (26)	
	Rarely or at least once a month	6 (32)	
	Occasionally	1 (5)	
	Weekly	3 (16)	
	Daily at least once a day	4 (21)	
**Has technology made your life easier, n (%)**			
	Easier	17 (89)	
	No difference	2 (11)	
	Harder	0 (0)	
**Do you think technology can help you take care of babies, n (%)**			
	Yes	19 (100)	
	No	0 (0)	
**How do you describe yourself, n (%)**			
	I am the first to try something new	6 (32)	
	Before I try, I watch others try it and see if it fits my life	13 (68)	
	I am usually among the last to try something new	0 (0)	
**How satisfied are you with the care of newborns at the Tororo District Hospital, n (%)**			
	1 (least satisfied)	1 (13)	
	2	2 (11)	
	3	8 (44)	
	4	2 (11)	
	5	5 (28)	
	6	0 (0)	
	7 (most satisfied)	0 (0)	
**How do you currently do the medication and fluid calculations, n (%)**			
	By handheld calculator	3 (16)	
	By calculator on the phone	11 (58)	
	I do them in my head	5 (26)	
**How do you decide when and which particular medication to give a sick baby, n (%)**			
	Check the World Health Organization chart at the maternity ward	14 (74)	
	Consult with the medical doctor	12 (63)	
	I use my judgment and experience	6 (32)	

### Adoption

**Figure 3:**
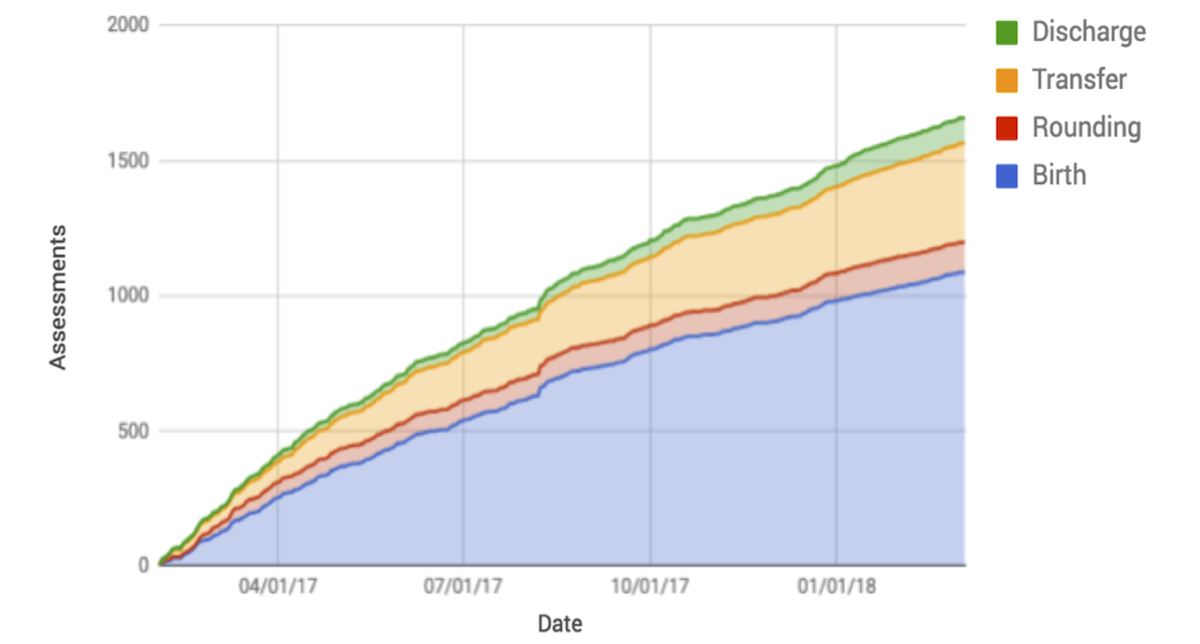
NoviGuide use by assessment type.

The study participants entered a total of 1705 assessments into NoviGuide over the study period. Of these 1705 assessments, 1412 (82.82%) were completed through to the summary page. The most common completed entries were birth assessments with 65.93% (931/1412) assessments for *new baby born in last 24 hours* and 20.25% (286/1412) assessments for *new baby more than 24 hours old or change in clinical status* (Figure 3), followed by *discharge* 6.51% (92/1412), *rounding* 5.59% (79/1412), seizure emergency 1.48% (21/1412), and abdominal emergency 0.21% (3/1412). Of the 293 uncompleted assessments, 161 (54.9%) were for *new baby born in last 24 hours,* 81 (27.6%) for new baby more than 24 hours old or change in clinical status, 32 (10.9%) for *rounding*, 2 (0.68%) for *discharge*, 15 (5.1%) for seizure emergency, and 2 (0.68%) for abdominal emergency.

The median time for a participant to complete assessments was 2.0 (IQR 1.0-5.0) min for *new baby born in last 24 hours*, 6.0 min (IQR 3.0-13.0) for *new baby more than 24 hours old or change in clinical status*, and 6.0 (IQR 4.0-10.5) min for *rounding*. In total, participants used the *My Patient* section for a total of 161 hours. NoviGuide was used frequently throughout the day, with 839 (49.2%) assessments made during the day shift, 700 (41.1%) during the evening shift, and 166 (9.7%) during the night shift (Table 3).

All but 1 of the 19 study participants recorded entries into the NoviGuide. The mean (range) number of completed assessments per study participant was 90 (0-321). The participant without entries had been transferred to another hospital shortly after enrolling. Of the 1092 assessments of babies born within the last 24 hours, 68.13% (744/1092) were completed by only 26% (5/19) study participants. Participants entered 46 practice cases, denoted by answering “N” (no) to the question, “Are you with a real baby? (Touch N if practicing).”

Data from the maternity register included 4704 admissions from February 1, 2017, to February 20, 2018. Of these, 97.55% (4589/4704) were identified as born at TDH, 2.32% (109/4704) were born outside of TDH, and 0.13% (6/4704) entries did not specify the birth site. Six deaths (0.13%) were recorded in the registry, and 0.64% (30/4704) newborns were transferred to a higher acuity facility. The registry, while noting whether the newborn was born at TDH, does not include the requisite data to determine whether the care encounter occurred immediately postpartum or upon return to the hospital following discharge.

**Table 3:** NoviGuide use by work shifts and time of day.

Assessment type	Assessments made during the different shifts and time of day				Total
	Day (8 AM to 2:59 PM)	Evening (3 PM to 7:59 PM)	Night (8 PM to 8 AM)		
New baby born in the last 24 hours, n	529	451	112	1092	
New baby more than 24 hours old, n	176	151	40	367	
Rounding, n	64	44	3	111	
Discharge, n	54	33	7	94	
Abdominal emergency, n	4	1	0	5	
Seizure emergency, n	12	20	4	36	
Total (%)	49.2	41.1	9.7	100	

### Acceptability

The overall SUS score at the end of the study was very high at 93.5 (Table 4) compared with the average score of 68, as described by John Brooke [30]. The mean (SD) scores of all the questions in the CHCE-PSQ were more than 4 (out of a maximum of 5; Table 5). The participants reported high levels of satisfaction with the NoviGuide (mean 4.86, SD 0.36). The participants’ perceptions about NoviGuide included the following: NoviGuide saved time (mean 5, SD 0), its information was useful (mean 4.79, SD 0.43), its information was easy to understand (mean 4.5, SD 0.52), the graphics were highly effective (mean 4.07, SD 0.47), and it could improve patient-nurse encounters (mean 5, SD 0).

In the end-of-study questionnaire (Table 5), the participants reported that NoviGuide helped them deliver better care and prevented them from making mistakes and that they felt more confident in taking care of newborns when they used the NoviGuide.

**Table 4:** Usability scores.

SUS^a^	Score, mean^b^ (SD)	Converted^c^
1. I think that I would like to use the NoviGuide frequently.	5 (0)	4
2. I found the NoviGuide unnecessarily complex.	1.14 (0.36)	3.9
3. I thought the NoviGuide was easy to use.	4.7 (0.46)	3.7
4. I think that I would need the support of a technical person to be able to use NoviGuide.	1.21 (0.43)	3.8
5. I found the various functions in the NoviGuide were well integrated.	4.86 (0.36)	3.9
6. I thought there was too much inconsistency in the NoviGuide.	1.36 (0.74)	3.6
7. I would imagine that most people would learn to use the NoviGuide very quickly.	4.57 (0.65)	3.6
8. I found NoviGuide very cumbersome to use.	1.21 (0.80)	3.8
9. I felt very confident using the NoviGuide.	5 (0)	4
10. I needed to learn a lot of things before I could get going with the NoviGuide.	1.86 (1.23)	3.1
Total converted mean scores × 2.5 (overall SUS score)	N/A^d^	93.5

^a^SUS: System Usability Scale.

^b^1: strongly disagree, 2: somewhat disagree, 3: neutral or no opinion, 4: somewhat agree, and 5: strongly agree.

^c^For items 1, 3, 5, 7, and 9, the converted score is the mean score minus 1. For items 2, 4, 6, 8, and 10, the converted score is 5 minus the mean score.

^d^N/A: not applicable.

**Table 5:** Mean scores of the Center for Health Care Evaluation Provider Satisfaction Questionnaire and the end-of-study questionnaire.

Questionnaires			Value, mean (SD)
**Center for Health Care Evaluation Provider Satisfaction Questionnaire^a^**			
	1. How useful is the information provided in the NoviGuide?	4.79 (0.43)	
	2. How easy is it to understand the information in the NoviGuide?	4.5 (0.52)	
	3. How effective are the graphics in NoviGuide?	4.07 (0.47)	
	4. What is your general satisfaction with the NoviGuide?	4.86 (0.36)	
	5. The NoviGuide could improve patient-nurse encounters	5 (0)	
	6. The NoviGuide saved me time	5 (0)	
	7. I would use it regularly in the clinic or hospital	4.71 (0.47)	
	8. I would recommend that other nurses use this tool	5 (0)	
**Acceptability: end-of-study questionnaire^b^**			
	1. The NoviGuide helped me deliver better care to newborns	5 (0)	
	2. The NoviGuide prevented me from making a mistake while providing care to newborns	5 (0)	
	3. The NoviGuide improved my documentation on newborns and mothers	4.79 (0.43)	
	4. I was proud to use the NoviGuide	4.93 (0.27)	
	5. I feel more confident taking care of newborns when I use the NoviGuide	5 (0)	
	6. I think that using NoviGuide made a good impression on parents of the newborns I have seen	4.71 (0.61)	
	7. I think that using NoviGuide made a good impression on other parents in the community	4.43 (0.65)	
	8. I think that NoviGuide improved newborn care at my hospital	4.93 (0.27)	
	9. I think that using the NoviGuide to deliver newborn care at other hospitals is a positive idea	5 (0)	
	10. I think that NoviGuide is an important part of meeting my needs in caring for newborns	5 (0)	
**Feasibility: end-of-study questionnaire**			
	11. I had the medical supplies and materials needed to follow NoviGuide recommendations	3.36 (1.22)	
	12. I had enough time to use the NoviGuide	4.57 (0.65)	
	13. My colleagues supported my use of the NoviGuide	4.71 (0.47)	
	14. My supervisor and the hospital administration supported my use of the NoviGuide	4.71 (0.47)	
	15. Technical support was always available for any difficulties I had with the NoviGuide	4.93 (0.27)	

^a^For Center for Health Care Evaluation Provider Satisfaction Questionnaire questions 1 to 4: 1, poor; 2, fair; 3, good; 4, very good; and 5, excellent; and for questions 5 to 8: 1, strongly disagree; 2, somewhat disagree; 3, neutral or no opinion; 4, somewhat agree; and 5, strongly agree.

^b^For the end-of-study questionnaire: 1, strongly disagree; 2, somewhat disagree; 3, neutral or no opinion; 4, somewhat agree; and 5, strongly agree.

### Feasibility

Study participants initiated and completed assessments on both well-appearing and ill-appearing newborns with diverse clinical characteristics (Table 6). Of the 1092 assessments for new baby born in last 24 hours, 29.21% (319/1092) were sick appearing, 24.82% (271/1092) had difficulty breathing, 19.51% (213/1092) weighed under 2.5 kg, 12.18% (133/1092) were born preterm, and 38.00% (415/1092) had at least one abnormal vital sign. Of the 367 assessments for new baby more than 24 hours old or change in clinical status, 77.4% (284/367) were sick appearing, 27.5% (101/367) had difficulty breathing, 53.1% (195/367) had at least one abnormal sign, 30.2% (111/367) weighed under 2.5 kg, 16.1% (59/367) were born preterm, and 59.9% (220/367) had antibiotics calculated during the assessment.

Rounding assessments included 6.51% (111/1705) of the total 1705 assessments entered into NoviGuide. Of these, 77.5% (86/111) were for *term baby needing treatment*, 18.0% (20/111) for *preterm baby*, and 4.5% (5/111) for *term baby*.

In a number of instances, participants working in the same maternity ward reported varying resource capabilities. In 738 assessments where participants were guided to check a glucose level, 46.3% (342/738) reported that a glucometer was available and entered a level, whereas 53.7% (396/738) responded *Cannot test*. Of the 264 completed assessments where a newborn had respiratory distress, participants indicated that a regular nasal cannula was available in 98.5% (260/264) assessments, whereas in 1.5% (4/264) assessments, participants indicated that there was no treatment available. Concerning the availability of IV fluids, participants indicated a desire to calculate IV fluid doses or rates in 328 assessments but indicated they could not give IV fluids in 20 assessments (6.1%).

In the end-of-study questionnaire, participants responded with a mean score of 3.3 (SD 1.22) when asked whether they had the requisite resources to follow NoviGuide’s recommendations (Table 5). The participants’ mean scores for the following questions were all above 4.5: (1) Was there enough time to use NoviGuide? (2) Was there support from their colleagues, supervisors, and hospital administration? and (3) Was technical support readily available?

**Table 6:** Characteristics of newborns entered into NoviGuide.

Newborn characteristics	New baby born in the last 24 hours (n=1092), percent birth assessments, n (%)	New baby more than 24 hours old (n=367), percent birth assessments, n (%)
Sick appearing	319 (29.21)	284 (77.4)
Difficulty in breathing	271 (24.82)	101 (27.5)
Weight under 2.5 kg	213 (19.51)	111 (30.2)
Preterm (<37 weeks)	133 (12.18)	59 (16.1)
Abnormal vital signs^a^	415 (38.00)	195 (53.1)
HIV exposed	55 (5.04)	12 (3.3)
Maternal fever	70 (6.41)	N/A^b^
Foul smelling amniotic fluid	109 (9.98)	N/A
Born in an unsterile environment	51 (4.67)	N/A
Antibiotics calculated during assessment	440 (40.29)	220 (59.9)

^a^Abnormal vital signs were defined as follows: temperature <36.5°C or >37.9°C, respiratory rate <30 or >60 breaths per minute, or heart rate <100 beats per minute or >160 beats per minute.

^b^N/A: not applicable.

### Sustainability

**Figure 4:**
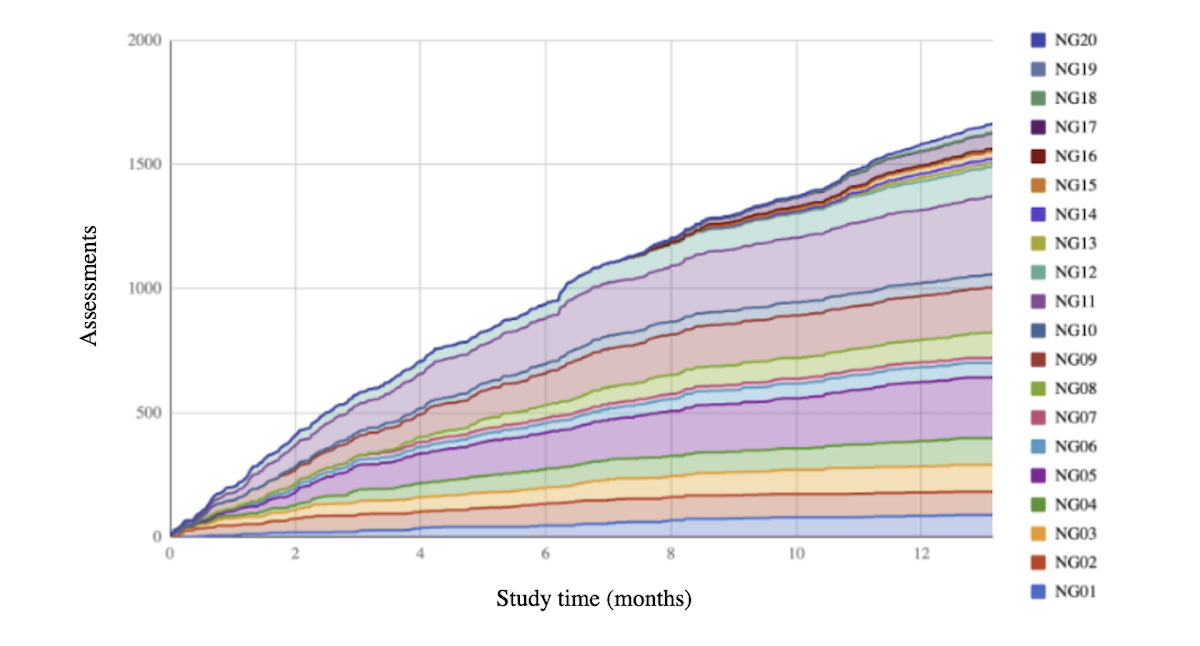
NoviGuide use by user. Individual study participants had unique identification numbers starting with NG, followed by the number, for example, NG01.

Although NoviGuide continued to be used regularly throughout the study (Figure 4), use declined with time. Study participants made 35.36% (603/1705) assessments on days 0-99 and then 27.74% (473/1705), 18.82% (321/1705), and 18.06% (308/1705) assessments over the subsequent 100-day intervals. There were only 3 instances of minor technical issues; the only technical issue reported was screen freezing, which was easily resolved by either the participant or the study team.

### Impact on Participant Knowledge

The results from the knowledge assessment questionnaires demonstrated significant improvement in basic newborn care knowledge over time. Among the 18 participants who were assessed at 6 months, scores increased from a mean of 10.4 to 14.1, reflecting a mean change of 3.7 (95% CI 2.6-4.8; *P*<.001) points. Among the 8 patients who were assessed at 12 months, the mean change from baseline was 6.7 (95% CI 5.07-8.31; *P*<.001).

## Discussion

### Principal Findings

NoviGuide was easily adapted to Uganda clinical guidelines, and its implementation in a rural district hospital was feasible and acceptable to nurse-midwives caring for newborns. The nurse-midwives used NoviGuide across a range of clinical scenarios, reported high levels of satisfaction with the software, and reported that it significantly improved their knowledge of newborn care. This study adds to the growing evidence that CDS software designed for facility-based health care workers delivering complex inpatient care can increase the use of national clinical guidelines in LMICs [32-35].

There are a number of features that distinguish NoviGuide from previously reported neonatal CDS software designed for LMICs [32,36-38]. NoviGuide converts guideline documents into patient-specific guidance, providing contextual drug dosing and cross-referencing diagnoses with vital sign inputs. Although other CDS apps only provide users with treatment guidance for a pathology the user has selected, NoviGuide not only provides treatment guidance but also prompts users to consider pathologies based on patient-specific inputs. Finally, NoviGuide is not a medical record by design and therefore does not require patient identification inputs. This makes the time required to complete an assessment through NoviGuide significantly shorter.

Ugandan experts suggested only two minor modifications to NoviGuide’s decision trees. Although the suggested additions are not explicitly detailed in the Uganda clinical guidelines, they do align with the national strategy to reduce deaths from newborn infections and with general standards of care. The paucity of modifications suggests that NoviGuide was well aligned to the Ugandan local context and suggests that there may be similar ease of adaptation in other countries where national guidelines are based on WHO recommendations on newborn health [36]. This finding has implications for scalability, as it suggests that algorithm templates with a few configurable settings may be acceptable in a wide range of health systems.

A key finding is that nurse-midwives had very high levels of satisfaction with NoviGuide; participants reported that the NoviGuide saved time, that they would recommend it to other nurses, and that they were even proud to use it. These findings highlight the potential of CDS as a delivery system for implementing complex clinical protocols. CDS-enabled functionalities, such as automated drug dose calculations, combined with a streamlined and attractive user interface, may confer a benefit on the user separate from that acquired by adhering to a specific clinical standard. Interestingly, these high levels of satisfaction with the NoviGuide persisted despite evidence that participants did not use it on every baby, there was wide variation in use, and there was an overall decrease in use over time. There are a number of possible explanations for the wide variation in use among participants, including differences in hours worked per individual, role within the ward, and planned absences. It is possible that autonomy in using, or not using, NoviGuide contributed to overall satisfaction with the software; NoviGuide may have been time saving because participants could self-select when they wanted to use it. Regardless, as in previous studies on CDS [20,37,38], our study demonstrates that the deployment of CDS in a way that does not mandate use will not capture all patients.

Participants improved their knowledge scores over the study period, even as they only rarely engaged with the parts of NoviGuide intended for self-directed learning outside of clinical care. This finding suggests that rather than becoming dependent on CDS to the detriment of internalized knowledge, CDS can improve provider knowledge through exposure. The finding that participants visited the self-directed learning section only 14 times over the study period requires additional qualitative investigation. One possibility is that health care providers form an early perception of the software as either a point-of-care software or a continuing education software, but not both.

A potentially important finding was that NoviGuide’s use data captured entries where participants working at the same hospital reported that they had different resources to treat patients. This finding has potentially important implications, as it suggests that CDS could be used to identify instances where either an individual or system barrier prevents available resources from being used. The same data, if transmitted frequently, could facilitate the rapid identification of resource gaps, such as the stock out of drugs or malfunction of a previously functioning medical equipment.

### Limitations

We acknowledge several limitations of our study. The newborn clinical characteristics entered into NoviGuide were not corroborated by reference to clinical charts or direct observation. Some of the investigators performing this evaluation were the designers of this tool, raising the possibility of bias; additional evaluation of our tool at a later stage in development could be informative. The lack of rolling enrollment may have influenced our adoption measurements, as new staff in the maternity ward began work before being enrolled in the study, resulting in a period where these staff observed NoviGuide in use but could not use it themselves. We also lack data to draw conclusions regarding the resource availability discrepancies identified through NoviGuide use. Specifically, we cannot determine whether these variances corresponded to a lack of provider comfort in using the resource, lack of access, equipment malfunction, or other causes. Finally, the Likert scale, which is commonly used to evaluate software acceptability and feasibility, is an imperfect tool and can result in *response style bias* [39].

### Conclusions

A CDS software for neonatal health care providers can be an alternative method for implementing complex neonatal protocols in LMICs and may improve upon, and complement, the standard didactic approach because of its ability to couple clinical protocols with job aide functionalities. The NoviGuide software was easily adapted to Uganda neonatal care clinical guidelines, was used across a range of clinical scenarios, resulted in high levels of satisfaction, and significantly improved knowledge among nurse-midwives. Although CDS is not a solution for all the training needs of a health care workforce in LMICs, it may be the optimal choice for content that is complex, not easily retained or applied, and for which immediate performance feedback is not possible.

## Appendix

Multimedia Appendix 1 NoviGuide resuscitation video.

Multimedia Appendix 2 NoviGuide step-by-step prompts.

Multimedia Appendix 3 System Usability Scale and provider satisfaction questionnaire adapted from the Center for Health Care Evaluation Provider Satisfaction Questionnaire, the end-of-study questionnaire, and the knowledge assessment questionnaire.

## Abbreviations

CDS: clinical decision support
CHCE-PSQ: Center for Health Care Evaluation Provider Satisfaction Questionnaire
IV: intravenous
LMIC: low- and middle-income country
PTBi: Preterm Birth Initiative
SUS: System Usability Scale
TDH: Tororo District Hospital
WHO: World Health Organization
