# Supplementary figures and images for: Implementation of a Newborn Clinical Decision Support Software (NoviGuide) in a Rural District Hospital in Eastern Uganda: Feasibility and Acceptability Study
Source: JMIR Mhealth Uhealth. 2021 Feb 19;9(2):e23737. doi: 10.2196/23737 (PMC7935651; doi:10.2196/23737)

## Slide 1
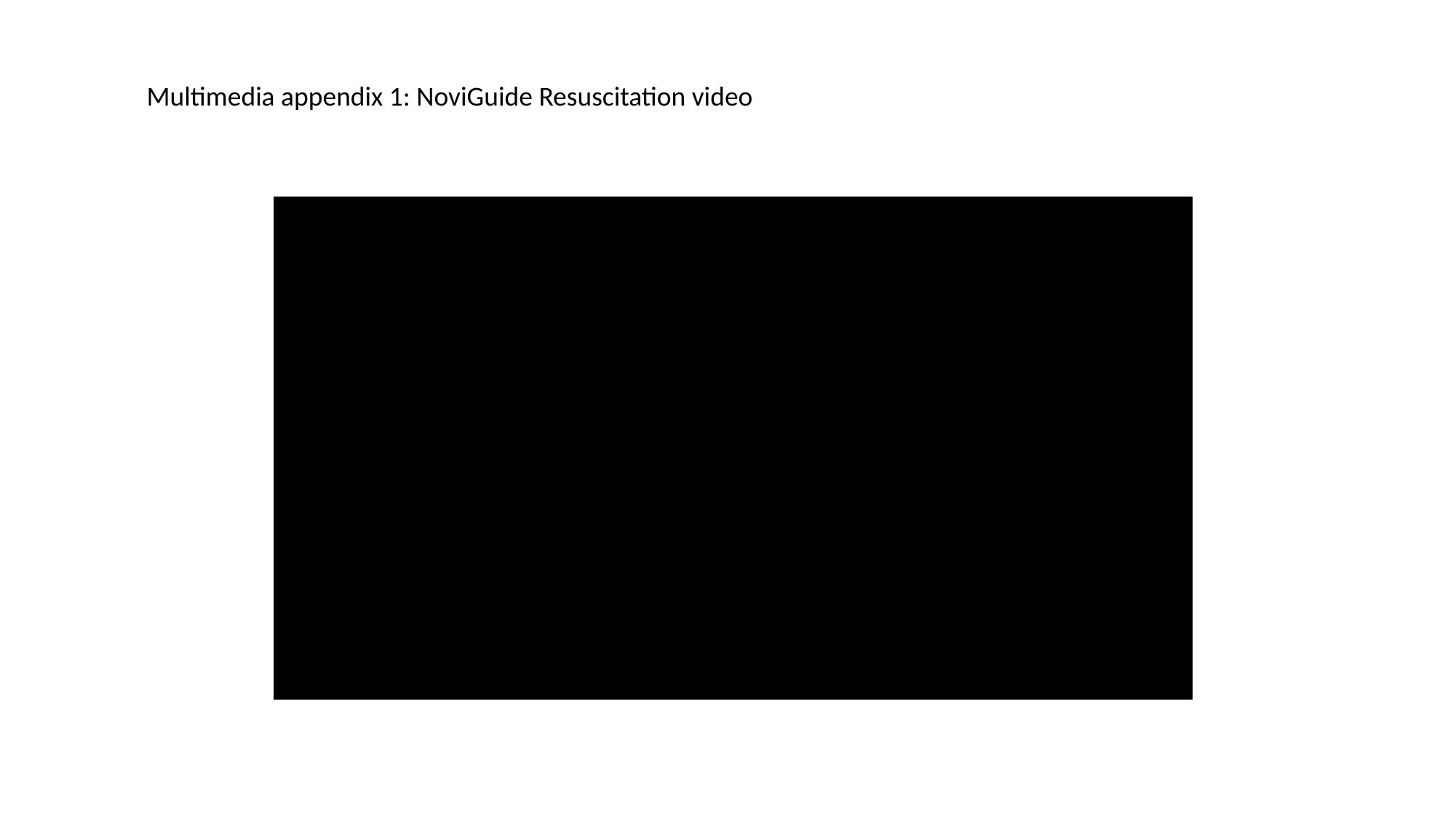

Multimedia appendix 1: NoviGuide Resuscitation video

Supplement: Multimedia Appendix 1 [file mhealth_v9i2e23737_app1.pptx]

## Slide 1
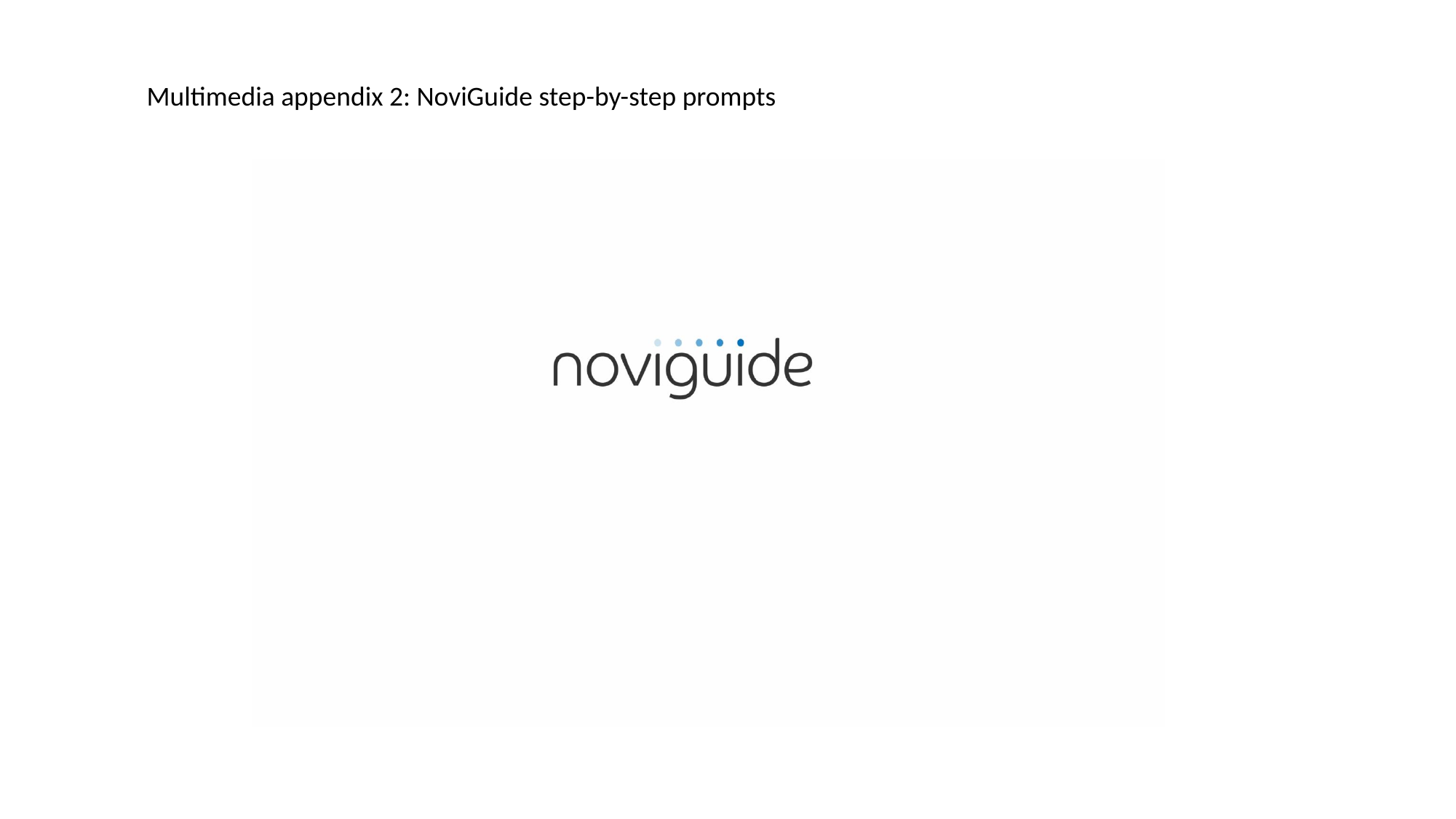

Multimedia appendix 2: NoviGuide step-by-step prompts

Supplement: Multimedia Appendix 2 [file mhealth_v9i2e23737_app2.pptx]
