# Supplementary material for: Implementation of a Newborn Clinical Decision Support Software (NoviGuide) in a Rural District Hospital in Eastern Uganda: Feasibility and Acceptability Study
Source: JMIR Mhealth Uhealth. 2021 Feb 19;9(2):e23737. doi: 10.2196/23737 (PMC7935651; doi:10.2196/23737)
Supplement: Multimedia Appendix 3 [file mhealth_v9i2e23737_app3.doc]

NoviGuide study BASELINE CHARACTERISTICS

***To be completed by participants at baseline.* The research assistant will check the box with the appropriate answer.**

1. **Demographics**
2. Sex:

^1^☐Female ^2^☐Male

1. Date of birth: /
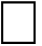
/

DAY MONTH YEAR

1. What language do you speak at home?

^1^☐English

^2^☐Luganda

^3^☐Japadhola

^4^☐ Ateso

^5^☐Other, ^Q3SPEC^specify______________________________________

1. **Clinical experience.**
2. Professional role:

^1^☐Nurse - Midwife

^2^☐Nursing Assistant

^3^☐Medical Officer

^4^☐Other, ^Q4SPEC^Specify____________________________

1. Highest Educational Degree Completed:

^1^☐Medical Doctor

^2^☐Doctorate (PhD)

^3^☐Master’s Degree

^4^☐Bachelors or College Degree

^5^☐Certified Midwife

^6^☐Physician Assistant (PA)

^7^☐Registered Nurse

^8^☐Licensed Practical nurse or Primary Nurse

^9^☐Other, ^Q5SPEC^Specify____________________________

1. Number of years of work experience:

^1^☐0-2 years

^2^☐3-10 years

^3^☐11-20 years

^4^☐21 plus years

1. Department(s) where you work (choose all that apply):

^7.1^☐Labor suite

^7.2^☐Post-partum ward

^7.3^☐Theatre

^7.4^☐General pediatrics wards

^7.5^☐Other, ^7.5spec^ please specify ______________________________

**Experience using technology**

1. Select all the device(s) you own personally. Select all that apply.

^1^☐None

^2^☐Home computer or laptop

^3^☐Tablet (e.g., iPad)

^4^☐Smart phone

^5^☐Other, ^Q9SPEC^specify ______________________________

1. How frequently do you access the internet?

^1^☐Never

^2^☐Rarely: less than once a month

^3^☐Occasionally: 1-3 times each month

^4^☐Weekly

^5^☐Daily: at least once a day

1. Has technology made your life easier or harder?

^1^☐Easier

^2^☐No difference

^3^☐Harder

1. Do you think technology can help you take care of babies?

^1^☐Yes

^2^☐No

^3^☐Not sure

1. How would you describe yourself?

^1^☐I am usually the first to try something new

^2^☐Before I try something new, I watch others try it and see if it fits into my life

^3^☐I am usually among the last to try something new

**Perceived challenges in caring for the newborn**

1. Approximately how many newborn babies (less than 28 days old) do you see per month (including births)?

^1^☐0

^2^☐1-10

^3^☐11-30

^4^☐31-100

^5^☐Greater than 100

1. Which type of children needing care do you regularly see at the TDH

^1^☐ Healthy, term babies

^2^☐Term babies needing treatment

^3^☐Preterm babies

^4^☐Babies born at home or in another facility

^5^☐Other, ^Q14SPEC^specify ______________________

1. How satisfied are you with the care of newborns at the TDH? **Circle 1 for Least Satisfied to 7 Most Satisfied**

**1 2 3 4 5 6 7**

1. When you encounter a preterm baby or sick child, what do you do:

^1^☐ I do not make any decisions on transfers, this decision is made by the doctor. I will continue to treat the baby until the decision is made.

^2^☐Refer them immediately to Mbale Hospital

^3^☐Stabilize the baby at TDH and then transfer once stable

^4^☐Treat the baby at TDH and refer only that treatment is unsuccessful

^5^☐Other, ^Q16SPEC^Specify ________________

1. How do you currently do medication and fluid dosing calculations?

^1^☐I don’t have to do medication calculations

^2^☐By hand

^3^☐By calculator

^4^☐By calculator on my phone

^5^☐I do them in my head

^6^☐Other, ^Q17SPEC^Specify _______________________________________

1. How do you decide when and which particular medication to give to a sick child

^1^☐Check the WHO chart at the maternity ward

^2^☐Consult with the medical doctor

^3^☐Guess

^4^☐I use my judgment and experience.

^5^☐Other, ^Q18SPEC^Specify _______________________________________

1. What state is the neonatal care at the TDH currently

^1^☐Doing well, has no problem

^2^☐Doing fairly well with some difficulties

^3^☐Not doing well at all and in need of significant improvement

1. What are your greatest challenges in caring for the newborn at the TDH?
2. ________________________________________________________
3. ________________________________________________________
4. ________________________________________________________

**Perceptions of the potential benefits of use of NoviGuide**

1. How do think NoviGuide will improve your ability to take care of babies, if at all?
2. How do you think the NoviGuide will improve your work, if at all?

**Anticipated challenges with the implementation.**

1. What challenges do you anticipate while using NoviGuide, if any?

**NOVIGUIDE STUDY** **Knowledge assessment questionnaire**

**INSTRUCTIONS:** This questionnaire is self-administered. You will need a pencil to complete the questionnaire. A calculator is recommended but not necessary.

WEIGHTS

1. You are visiting a term baby born to a first time mother who had a caesarian section for breech presentation. It is now day of life 3 (birth = day of life 0). The baby was born at 3.150kg (kilograms). Today the baby weighs 2.700kg. You ask the mother how the baby is feeding and the mother says “I think it is going okay, but I’m not sure.” Given this limited information, which of the following statements is true? Select one.

1

2

3

- All babies gain weight in the first 3 days of life. The fact that the baby lost weight is abnormal.
- Babies lose weight in the first 3 days of life and this amount of weight loss is normal.
- Babies lose weight in the first 3 days of life, but this amount of weight loss is more than expected.

2. You are visiting a baby born by vaginal delivery at 37 weeks gestation. The baby’s birthweight was 2.65kg. Today is now day of life 1 (birth = day of life 0). The baby is weighed again and now weighs 3.00kg. The baby is healthy appearing, breastfeeding and not receiving IV fluids. Given this limited information, which of the following statements is true? Select one.

- All babies gain weight in the first 3 days of life, so this is normal and expected.

1

2

3

- It is unlikely that a baby could gain this amount of weight in 1 day. Likely one of the two weights is incorrect.
- It is not common to have weight gain in the first 3 days of life, but if the mother had a lot of milk it is possible to gain a lot of weight in 24 hours.

VITAL SIGNS

3. You are evaluating a baby born at 35 weeks gestation after a rapid vaginal delivery. The baby’s mother had a fever to 38.7 degrees Celsius at the time of delivery. When you evaluate the baby, his respiratory rate is 90 breaths per minute and he has nasal flaring and retractions. The baby’s temperature is 38.9 degrees Celsius. You and your team are trying to decide what to do first for this baby. Given this limited information, which of the following statements is true? Select one.

- The respiratory distress should be addressed first.

1

2

3

- The baby needs antibiotics and those antibiotics should be given before addressing the respiratory distress.
- The respiratory rate is normal, so antibiotics should be given first.

4. You are evaluating a baby brought in after a home delivery. You take the vital signs. The vital signs show a respiratory rate of 55 breaths per minute, a heart rate of 215 beats per minute and a temperature of 37.1. Which of the following statements is true? Select one.

- Only the heart rate is abnormal.

1

2

3

- Only the respiratory rate is abnormal.
- Both the heart rate and respiratory rate are abnormal.

5. You are evaluating a healthy baby born yesterday by vaginal delivery at 40 weeks gestation. The temperature is 36.1 degrees Celsius. The mother reports everything is going very well and that the baby has been breastfeeding like her other children. Which of the following statements is true? Select one.

1

2

3

- - 36.1 is a normal temperature in a baby.
  - 36.1 is a low temperature, but because the mother is experienced, this is likely fine.
  - 36.1 is a low temperature and this needs to be addressed.

GLUCOSE

6. You have been called to evaluate a baby who is jittery. The mother says the baby is tired and does not want to eat. The baby is a 2.31kg baby born at 38 weeks gestation by vaginal delivery. The mother had gestational hypertension. You are worried about a low glucose, but do not have a glucometer. The baby is put on the breast, but is too tired to feed. Which of the following is an appropriate next step? Select one.

- - Continue to watch the baby closely.

1

2

3

4

- - Place an IV and give 4.6ml of D10% IV Dextrose as a bolus followed by a continuous infusion of IV Dextrose.
  - Place an IV and give 40ml of D10% IV Dextrose as a bolus.
  - Place an IV and give 10ml/kg of NORMAL SALINE.

7. You are evaluating a baby 1 hour after birth who was born at 4.45kg by c-section at 41 weeks gestation. You check the baby’s glucose and it is 3.6mmol/L (65 mg/dl). Which of the following is true? Select one.

- - This is a normal glucose.

1

2

3

- - This glucose is low and the baby should eat now if possible.
  - This baby has no risk factors for low glucose and therefore the glucose should not have been checked. Continue to follow the baby.

8. You are evaluating a baby 1 hour after birth who was born at 2.31kg by vaginal delivery at 36 weeks gestation. You check the baby’s glucose and it is 1.1 mmol/L (20 mg/dl). Which of the following is true? Select one.

- - This is a normal glucose for a small baby.

1

2

3

- - This glucose is very low and should be treated with IV Dextrose immediately.
  - The glucose is low, but not very low. When the mother is ready, she can breastfeed the baby.

9. You are evaluating a baby 4 hours after birth who was born at 3.1 kg by vaginal delivery at 38 weeks gestation. The baby is jittery and lethargic. Which of the follow is true? Select one.

1

2

3

- - Term babies with normal weights do not have low glucose levels. The symptoms are likely not related to the baby’s glucose.
  - The baby should have the glucose checked immediately.
  - These symptoms are abnormal, but do not require immediate treatment. Check on the baby again in 30 minutes.

INFECTION

10. You are attending a delivery of a 24 year old mother. The mother had a rupture of membranes (bag of water broke) three days ago. When she presented to the hospital, the mother had a temperature of 38.5 degrees Celsius. The amniotic fluid had meconium and was also foul smelling. When the baby was born, she was vigorous and crying with Apgar scores of 9,9. The baby is well appearing and interested in breastfeeding. Which of the following statements is true? Select one.

- - This baby has multiple risk factors for infection. According to the World Health Organization and Ugandan guidelines, the baby should be started on antibiotics even though she is well appearing.

1

2

3

- - This baby has multiple risk factors for infection. However, because she is well appearing, the World Health Organization and Ugandan guidelines do not recommend treatment with antibiotics. Observe the baby closely.
  - Though it is abnormal for the mother to have fever, it is normal to have the bag of water ruptured for 3 days and the meconium in the amniotic fluid is a normal finding. There are not enough risk factors to consider the baby “at risk” for infection.

11. You are rounding on a baby born yesterday by vaginal delivery. The baby is cold, lethargic and feeding poorly. You are concerned about neonatal sepsis and decide to start antibiotics. Which of the following combinations, when given together, are appropriate treatments for neonatal sepsis? Select all that apply.

- Metronidazole and Ampicillin

1

2

3

4

5

- Ampicillin and Gentamicin
- Ampicillin and Penicillin G
- Penicillin G and Gentamicin
- Cefotaxime and Gentamicin

12. You are caring for a set of twins being treated with gentamicin. The twins were born at 38 weeks gestation. The twins are not the same size. Twin A is 2.61kg. Twin B is 1.83kg. Calculate the dose in milligrams of gentamicin that you would give each baby.

13. You are caring for a premature baby being treated for neonatal sepsis. The baby was born to a mother with a fever to 39.2 degrees Celsuis. The baby was ill appearing after being born and was started on ampicillin and gentamicin. According to the World Health Organization, how long should you treat this baby with antibiotics (you can provide either a number of days or a range of days in your answer)?

HIV

14. You deliver the baby of a mother who is HIV positive. The mother has been followed in the prenatal clinic and has been taking her antiretroviral medication regularly. The baby is born by vaginal delivery and is vigorous and healthy. The mother did not have a fever prior to delivery. The membranes were ruptured near the time of delivery and the amniotic fluid was clear. Regarding the treatment of the baby, which of the following is true? Select one.

- Because the mother is on antiretroviral therapy, the baby is low risk and does not need medication.

1

2

3

- The baby should receive liquid nevirapine beginning shortly after birth.
- The baby should receive liquid nevirapine, but should also be treated with antibiotics because all babies born to HIV positive mothers are at high risk of infection.

15. A mother arrives at the hospital and immediately delivers a 3.45kg healthy term baby. When you review her records, you see that she was never tested for HIV. Which of the following is the most appropriate next step? Select one.

- Tell the mother she should get the test after she goes home. It is important for her to know her status.

1

2

3

- The mother should be tested immediately.
- Because the baby is healthy and has a normal weight, it is unlikely that he was born to an HIV positive mother. This baby most likely does not have HIV.

Feeding and IV Fluids

16. A mother walks into the labor & delivery and promptly delivers a 1.54kg baby who appears premature. You estimate that the baby is 32 weeks gestation. The baby is in respiratory distress and you are treating the respiratory distress with a nasal cannula. The baby is breathing 90 times per minute and has retractions. Do you think this baby needs IV fluids? Select one.

- Yes

1

2

- No

17. You are caring for a baby born at 29 weeks gestation who is now 7 days old. The mother has had 5 children. She asks you, “Can I breastfeed my baby?” Which of the following is the most appropriate response? Select one.

- Yes, breastmilk is the best nutrition for the baby.

1

2

3

4

- Yes, but the baby is premature and will need to be watched closely. It is okay to breastfeed, but follow the baby’s weight closely and if the weight drops, then place a nasogastric tube.
- The baby needs breastmilk, but is unlikely to be able to eat by mouth. The milk should be given by a nasogastric tube and the baby should attempt breastfeeding when she is more mature.
- This baby is too premature to receive breast milk. This baby should only be receiving IV fluids.

18. You are caring for a baby who is receiving IV fluids because he is too sick to eat. He was born by c-section and had respiratory distress at birth. The baby weighs 3kg. The IV fluid is going into the baby at 40ml/hr (using pediatric IV tubing, 40 drops per minute). Given this information, which of the following is true? Select one.

1

2

3

- This is an appropriate amount of IV fluids for a sick baby.
- The IV fluid rate is likely too slow and should be increased.
- The IV fluid rate is likely too fast and should be decreased.

RESUSCITATION

19. You deliver a baby born with the umbilical cord around the neck. The baby is blue with poor tone and is not breathing. You bring the baby to the resuscitation area. When should you begin to provide positive pressure ventilation with the bag and mask? Please provide a length of time in either minutes or seconds.

20. A mother arrives at the hospital because she felt that the baby was not moving inside her. A c-section is done urgently and the baby is delivered. The baby is blue with poor tone and is not breathing. You are providing positive pressure ventilation with the bag and mask. It is now 45 seconds since the baby was born. You look at the baby’s chest and see that the chest is not rising. Which of the following is the most appropriate next step? Select one.

- Begin chest compressions.

1

2

3

4

- Deliver adrenaline (epinephrine) via an IV.
- Reposition the mask and confirm a good seal. Then continue to provide positive pressure ventilation and verify that the chest is now rising.

Vigorously stimulate the baby by rubbing the baby’s head

**NoviGuide Study: 12 Month Self-Administered Provider Questionnaire**

**PART ONE**

**Instructions:** Please circle the number that corresponds to your opinions about each of the statements below.

1. I think that I would like to use the NoviGuide frequently.

1 – Strongly disagree 2 – Somewhat disagree 3 – Neutral/no opinion 4 – Somewhat agree 5 – Strongly agree

1. I found the NoviGuide unnecessarily complex.

1 – Strongly disagree 2 – Somewhat disagree 3 – Neutral/no opinion 4 – Somewhat agree 5 – Strongly agree

1. I thought the NoviGuide was easy to use.

1 – Strongly disagree 2 – Somewhat disagree 3 – Neutral/no opinion 4 – Somewhat agree 5 – Strongly agree

1. I think that I would need the support of a technical person to be able to use NoviGuide.

1 – Strongly disagree 2 – Somewhat disagree 3 – Neutral/no opinion 4 – Somewhat agree 5 – Strongly agree

1. I found the various functions in the NoviGuide were well integrated.

1 – Strongly disagree 2 – Somewhat disagree 3 – Neutral/no opinion 4 – Somewhat agree 5 – Strongly agree

1. I thought there was too much inconsistency in the NoviGuide.

1 – Strongly disagree 2 – Somewhat disagree 3 – Neutral/no opinion 4 – Somewhat agree 5 – Strongly agree

1. I would imagine that most people would learn to use the NoviGuide very quickly.

1 – Strongly disagree 2 – Somewhat disagree 3 – Neutral/no opinion 4 – Somewhat agree 5 – Strongly agree

1. I found NoviGuide very cumbersome to use.

1 – Strongly disagree 2 – Somewhat disagree 3 – Neutral/no opinion 4 – Somewhat agree 5 – Strongly agree

1. I felt very confident using the NoviGuide.

1 – Strongly disagree 2 – Somewhat disagree 3 – Neutral/no opinion 4 – Somewhat agree 5 – Strongly agree

1. I needed to learn a lot of things before I could get going with the NoviGuide.

1– Strongly disagree 2 – Somewhat disagree 3 – Neutral/no opinion 4 – Somewhat agree 5 – Strongly agree

**PART TWO**

**Instructions:** Please circle the appropriate number that best describes your opinion about NoviGuide.

1. How useful is the information provided in the NoviGuide?

1– Poor 2 – Fair 3 – Good 4 – Very Good 5 – Excellent

1. How easy is it to understand the information in the NoviGuide?

1– Poor 2 – Fair 3 – Good 4 – Very Good 5 – Excellent

1. How effective are the graphics in NoviGuide?

1– Poor 2 – Fair 3 – Good 4 – Very Good 5 – Excellent

1. What is your general satisfaction with the NoviGuide?

1– Poor 2 – Fair 3 – Good 4 – Very Good 5 – Excellent

**Instructions:** Please circle the appropriate number that corresponeds to your opinions about each of the statements below.

1. The NoviGuide could improve patient-nurse encounters

1 – Strongly disagree 2 – Somewhat disagree 3 – Neutral/no opinion 4 – Somewhat agree 5 – Strongly agree

1. The NoviGuide saved me time

1 – Strongly disagree 2 – Somewhat disagree 3 – Neutral/no opinion 4 – Somewhat agree 5 – Strongly agree

1. I would use it regularly in the clinic or hospital

1 – Strongly disagree 2 – Somewhat disagree 3 – Neutral/no opinion 4 – Somewhat agree 5 – Strongly agree

1. I would recommend that other nurses use this tool

1 – Strongly disagree 2 – Somewhat disagree 3 – Neutral/no opinion 4 – Somewhat agree 5 – Strongly agree

**NOVIGUIDE STUDY** End of Study Questionnaire 12 months

**INSTRUCTIONS:** Please circle the appropriate number that corresponds to your opinions about each of the statements below.

*Acceptability*

1. The NoviGuide helped me deliver better care to newborns

1 – strongly disagree 2 – somewhat disagree 3 – neutral/no opinion 4 – somewhat agree 5 – strongly agree

1. The NoviGuide prevented me from making a mistake while providing care to newborns

1 – strongly disagree 2 – somewhat disagree 3 – neutral/no opinion 4 – somewhat agree 5 – strongly agree

1. The NoviGuide improved my documentation on newborns and mothers

1 – strongly disagree 2 – somewhat disagree 3 – neutral/no opinion 4 – somewhat agree 5 – strongly agree

1. I was proud to use the NoviGuide

1 – strongly disagree 2 – somewhat disagree 3 – neutral/no opinion 4 – somewhat agree 5 – strongly agree

1. I feel more confident taking care of newborns when I use the NoviGuide

1 – strongly disagree 2 – somewhat disagree 3 – neutral/no opinion 4 – somewhat agree 5 – strongly agree

1. I think that using NoviGuide made a good impression on parents of the newborns I’ve seen

1 – strongly disagree 2 – somewhat disagree 3 – neutral/no opinion 4 – somewhat agree 5 – strongly agree

1. I think that using NoviGuide made a good impression on other parents in the community

1 – strongly disagree 2 – somewhat disagree 3 – neutral/no opinion 4 – somewhat agree 5 – strongly agree

1. I think that NoviGuide improved newborn care at my hospital

1 – strongly disagree 2 – somewhat disagree 3 – neutral/no opinion 4 – somewhat agree 5 – strongly agree

1. I think that using the NoviGuide to deliver newborn care at other hospitals is a positive idea

1 – strongly disagree 2 – somewhat disagree 3 – neutral/no opinion 4 – somewhat agree 5 – strongly agree

1. I think that NoviGuide is an important part of meeting my needs in caring for newborns

1 – strongly disagree 2 – somewhat disagree 3 – neutral/no opinion 4 – somewhat agree 5 – strongly agree

*Feasibility*

1. I had the medical supplies and materials I needed to follow the information presented in NoviGuide

1 – strongly disagree 2 – somewhat disagree 3 – neutral/no opinion 4 – somewhat agree 5 – strongly agree

1. I had enough time to use the NoviGuide

1 – strongly disagree 2 – somewhat disagree 3 – neutral/no opinion 4 – somewhat agree 5 – strongly agree

1. My colleagues supported my use of the NoviGuide

1 – strongly disagree 2 – somewhat disagree 3 – neutral/no opinion 4 – somewhat agree 5 – strongly agree

1. My supervisor and the hospital administration supported my use of the NoviGuide

1 – strongly disagree 2 – somewhat disagree 3 – neutral/no opinion 4 – somewhat agree 5 – strongly agree

1. Technical support was always available for any difficulties I had with the NoviGuide

1 – strongly disagree 2 – somewhat disagree 3 – neutral/no opinion 4 – somewhat agree 5 – strongly agree
